# Supplementary material for: Single-cell RNA-seq reveals fibroblast heterogeneity and increased mesenchymal fibroblasts in human fibrotic skin diseases
Source: Nat Commun. 2021 Jun 17;12:3709. doi: 10.1038/s41467-021-24110-y (PMC8211847; doi:10.1038/s41467-021-24110-y)
Supplement: Supplementary file 1 — Supplementary Information [file 41467_2021_24110_MOESM1_ESM.pdf]

Supplementary Materials
Supplementary Fig. 1

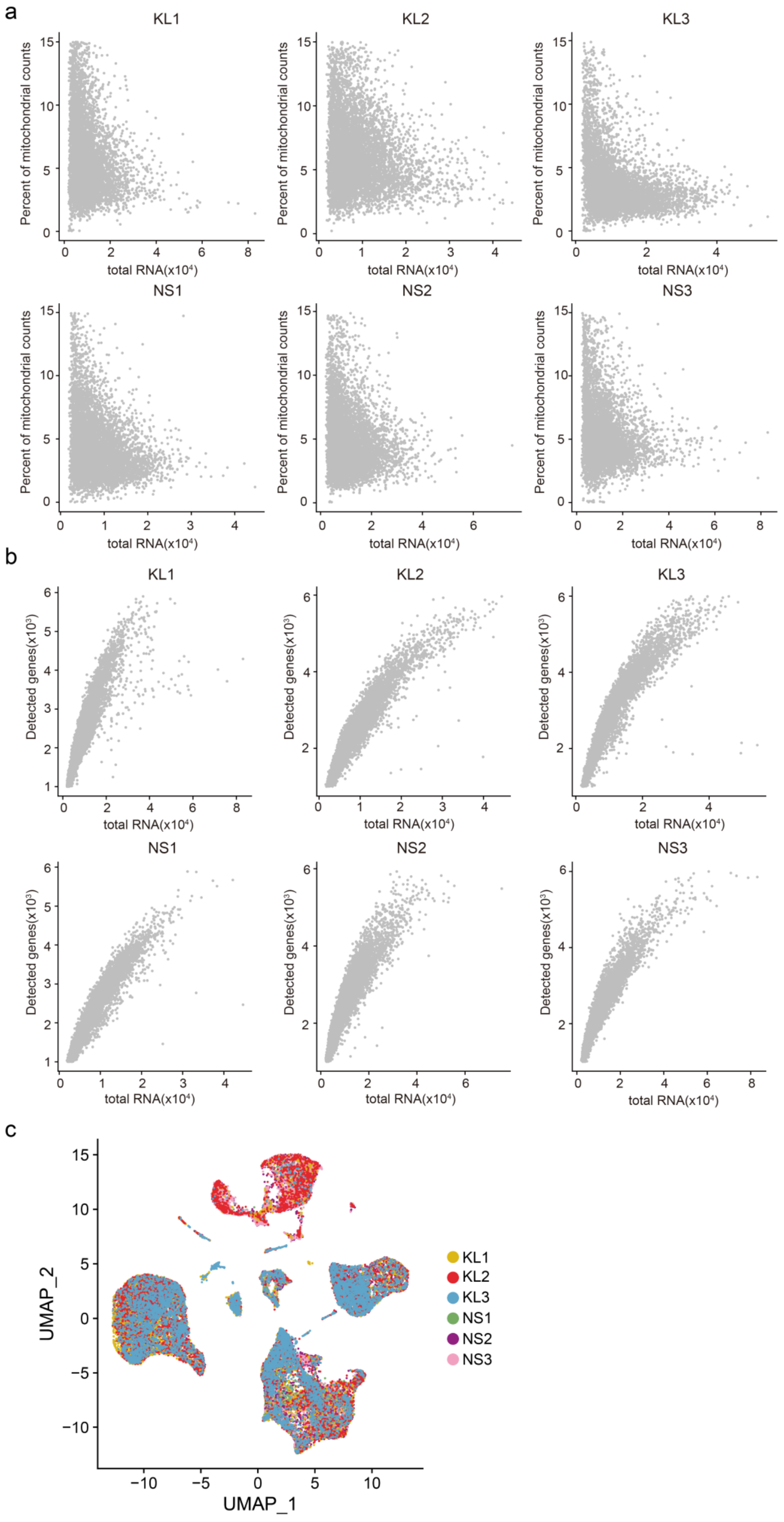

Supplementary Fig. 1. Quality control of all cells acquired by scRNA-seq of human keloid (KL) and normal scars (NS) samples. (a-b) Scatter plots show the quality control metrics used in the scRNAseq analysis. The percentage of reads that map to the mitochondrial genome was shown in (a), and the number of unique genes detected in each cell was shown in (b). **(c)** UMAP plot of all cells acquired by scRNA-seq of human keloid and normal scars samples. Cells obtained from different samples were labeled in different colors.

Supplementary Fig. 2

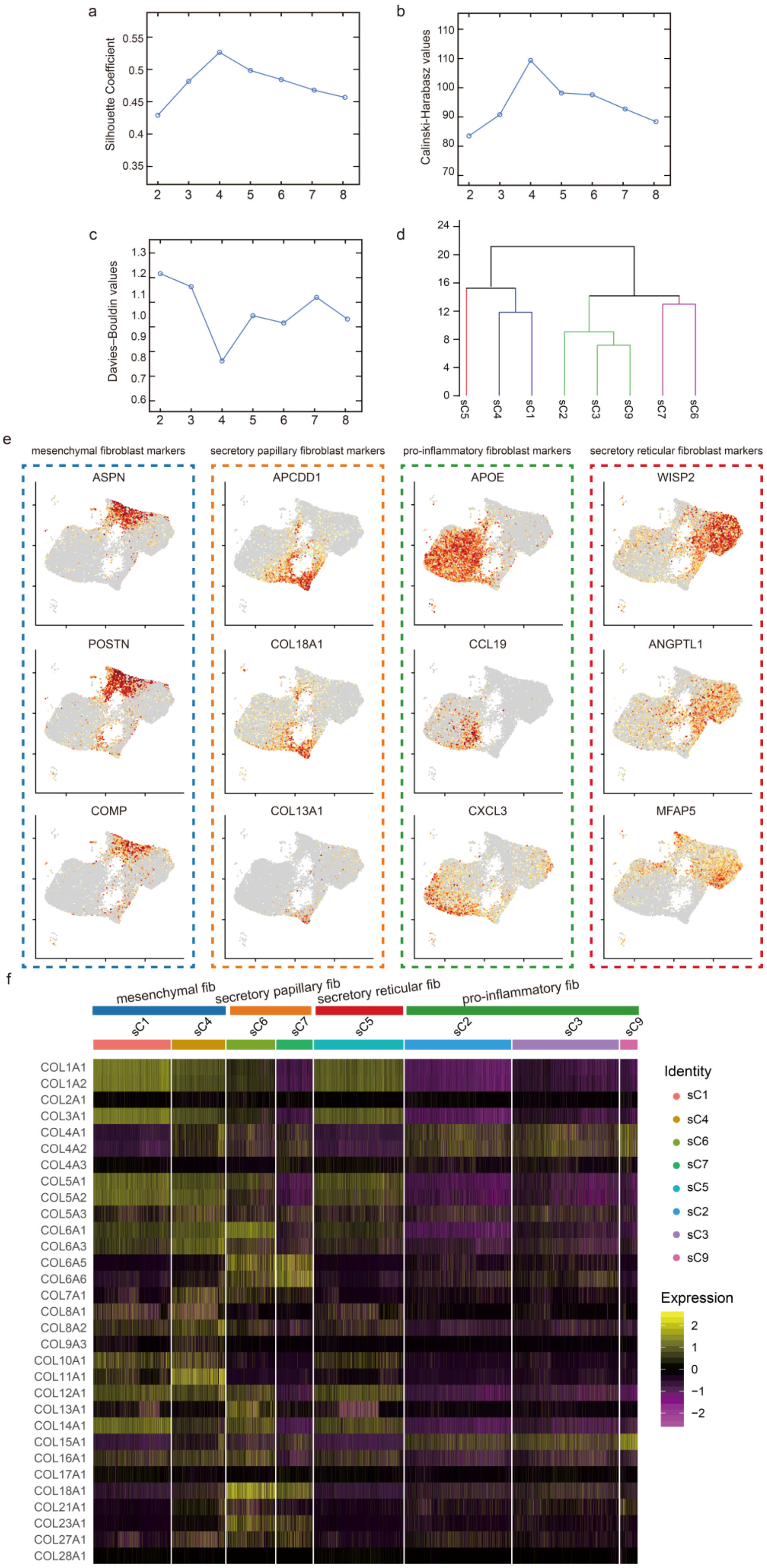

Supplementary Fig. 2. The analyses of the optimal number of clusters for the hierarchical-clustering solution in Figure 2d. (a) The silhouette coefficient, calculated for clusters 2 to 8. The maximum achieved values indicated the best clustering of the data. **(b)** The Calinski-Harabasz index, calculated for clusters 2 to 8. The maximum achieved index values indicated the best clustering of the data. **(c)** The Davies–Bouldin index, calculated for clusters 2 to 8. A lower value will mean that the clustering is better. **(d)** The dendrogram for hierarchical clustering. The y-axis represents the distance between clusters. Various colors represent the four-cluster solution chosen. **(e)** Feature plots of representative marker genes of each fibroblast subpopulation. Expression levels for each cell are color-coded and overlaid onto UMAP plot. **(f)** Heatmap showing the expression of all collagen genes in the distinct fibroblast subgroups. Each column represents a single cell and each row an individual collagen gene. Yellow indicates maximum gene expression while purple indicates no expression in scaled log-normalized UMI counts.

Supplementary Fig. 3

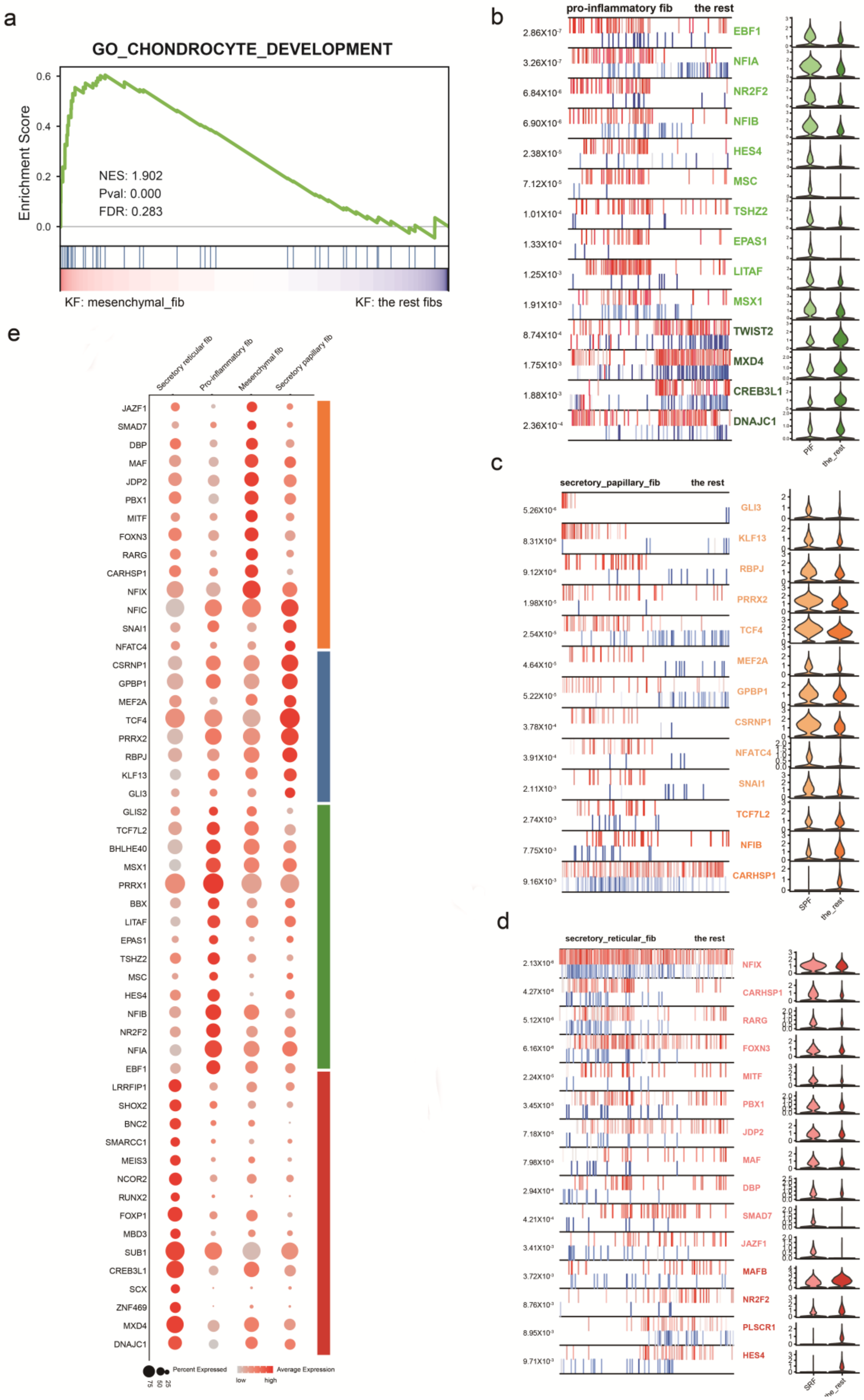

Supplementary Fig. 3. GSEA analysis and master transcription factor regulators analysis of four types of fibroblasts in keloid. (a) GSEA analysis for upregulated pathways in mesenchymal fibroblasts (NES normalized enrichment score, corrected for multiple comparisons using FDR method, *P* value were showed in plots). **(b-d)** The candidate master transcription factors (TFs) for each fibroblast subpopulation identified by an algorithm for master regulator analysis algorithm (MARINa). MARINa plots show activated targets colored red and repressed targets colored blue for each potential master TFs (vertical lines on the x axis). On the x axis, genes were rank-sorted by their differential expression in the chosen fibroblast subpopulation and the rest subpopulations. The *P*-values on the left indicate the significance of enrichment, calculated by permutating the chosen fibroblast subpopulation and the rest subpopulations. **(e)** Dot plots showing expression of the candidate master transcription factors in each fibroblast subpopulation. The area of the circles indicates the proportion of cells expressing the gene, and the color intensity reflects the expression intensity.

Supplementary Fig. 4

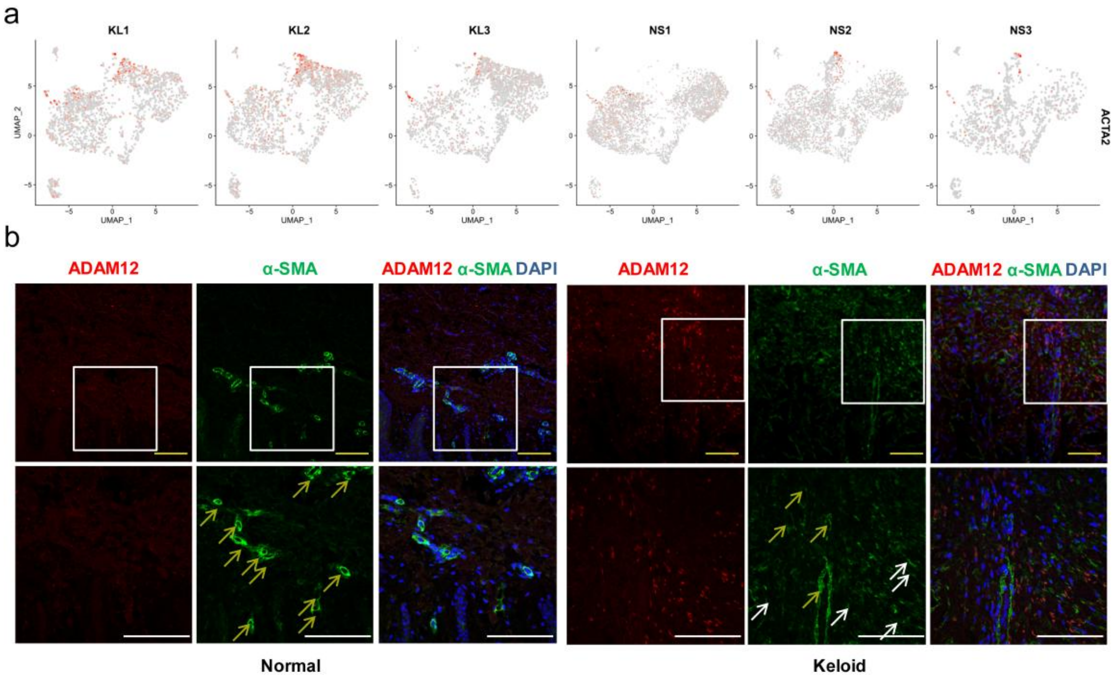

Supplementary Figure 4. Most of myofibroblasts were in the mesenchymal fibroblasts subpopulation. (a) Feature plots of expression distribution for ACTA2 in keloid fibroblasts (KL) and normal scar fibroblasts (NS). Expression levels for each cell are color-coded and overlaid onto the UMAP plot. **(b)** Immunofluorescence staining of ADAM12 and α-SMA (encoded by ACTA2) in normal and keloid tissues. Lower panels are the insets of upper panels. White arrowheads indicate myofibroblasts, and yellow arrowheads indicate vascular smooth muscle cells. Scale bar=200μm. Here showing representative images from 3 normal tissues and 3 keloid tissues.

Supplementary Fig. 5

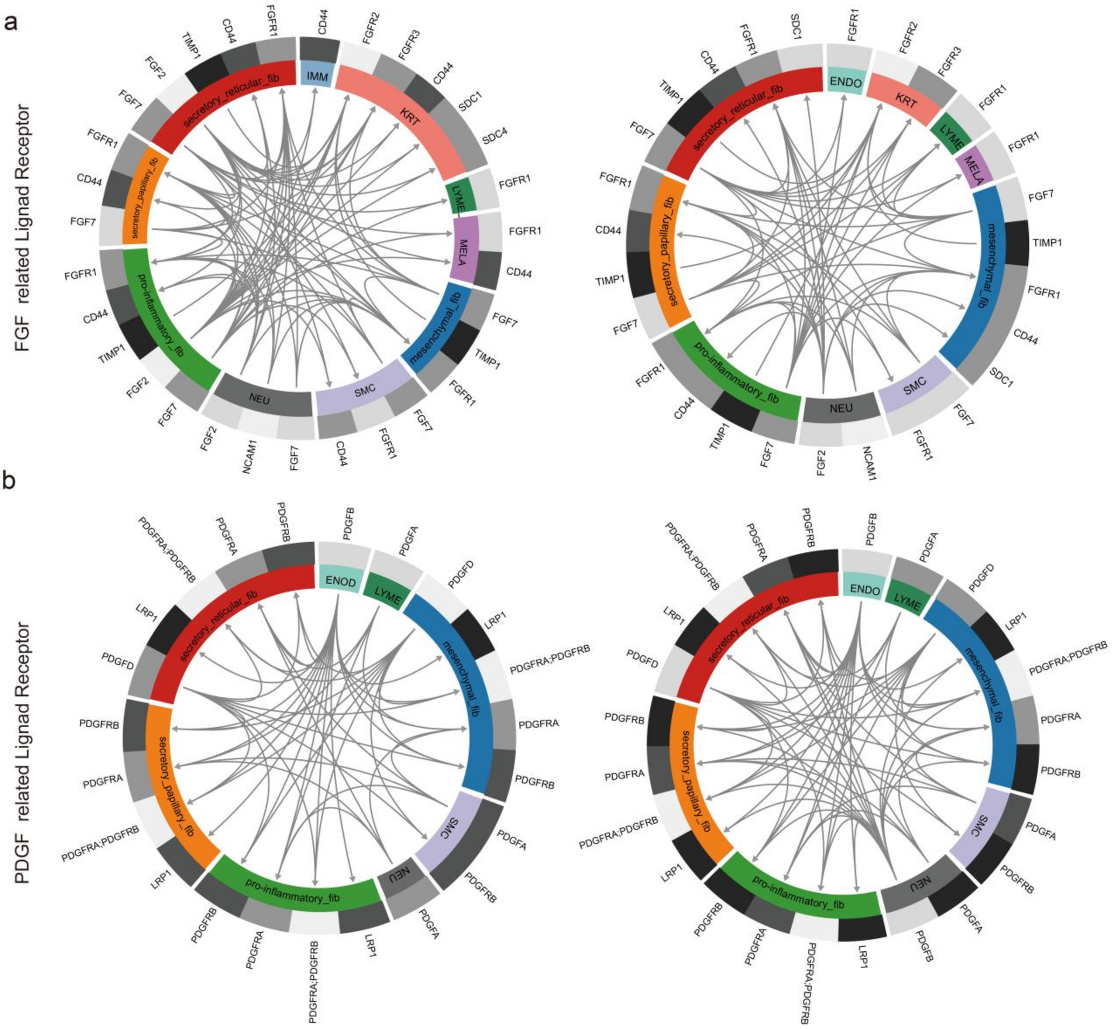

Supplementary Fig. 5. Putative FGF and PDGF related signaling within fibroblasts and other cell populations. (a-b) FGF (a) and PDGF (b) signaling Inter-lineage communication networks in normal scar tissues (left panel) and keloid (right panel). The line thickness is proportional to the number of broadcast ligands. The dot size reflects the number of interactions of the cell lineages.

Supplementary Fig. 6

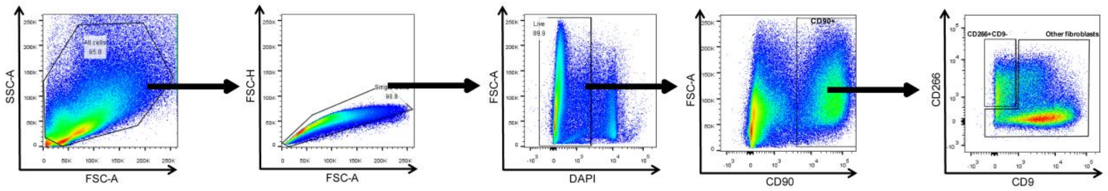

Supplementary Fig. 6. The gating strategy of Figure 5a.

Supplementary Fig. 7

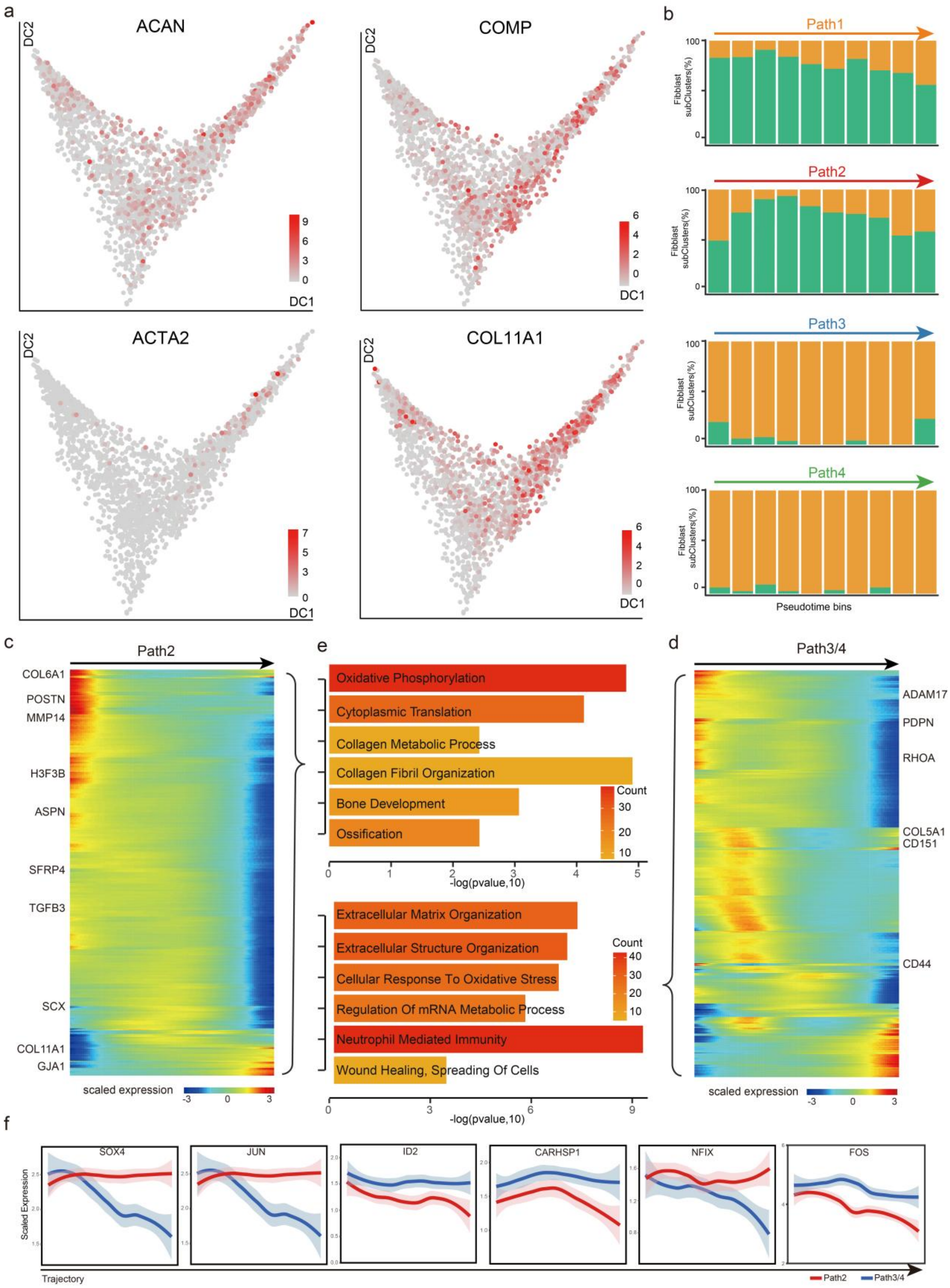

Supplementary Fig. 7. sC4 fibroblasts are more mesenchymal-like than sC1 fibroblasts. **(a)** Feature plots of expression distribution for ACAN, COMP, ACTA2 and COL11A1 across pseudotime. Expression levels for each cell are color-coded. Red indicates maximum relative expression and grey indicates low or no expression of the gene. **(b)** Stacked bar charts show relative abundance of fibroblast subclusters across individual RNA velocity paths. Fibroblast subcluster colors match the colors in Fig. 6A. **(c-d)** Heatmaps of pseudotime-dependent gene expression in RNA velocity Path2 (c) and RNA velocity Path3/4 (d). **(e)** Functional enrichment showing that distinct biological process related to RNA velocity Path2 cells and RNA velocity Path3/4 cells. **(f)** Smoothed expression of most differnetial expression of tanscription factors along RNA velocity Path2 and RNA velocity Path3/4. Normalized expressions are plotted against the pseudotime axis, and the gray shading indicates mean values ± 99% confidence interval (CI).

Supplementary Table 1. Patient demographics and characteristics.

Tissue collected	No. samples	Age (y)	Gender	Race	Site of collection	Intralesional steroids prior to excision
normal scar	3	39	M	Han nationality	back	no
		28	M	Han nationality	chest	no
		26	F	Han nationality	chest	no
keloid	3	20	M	Han nationality	back	no
		23	M	Han nationality	chest	no
		34	F	Han nationality	chest	no

Supplementary Table 2. The primers used in real-time quantitative PCR.

Symbol	Forward primer (5’→3’)	Reverse primer (5’→3’)
POSTN	GAGACAAAGTGGCTTCCG	CTGTCACCGTCACATCCT
COMP	AGCGAGTGCCACGAGCAT	CGGGAAGCCGTCTAGGTCA
ADAM12	GACTACAACGGGAAAGCAA	GAGCGAGGGAGACATCAGTA
COL11A1	AAGGCTCAGATACTGCTTACA	CTCCCAACCTCAACACCA
ASPN	CAGTCCCAACCAACATTC	GGACAGATACAGCCTTCG
P311	GGTTTATTACCCAGAACTCTTT	CAGCGTTTGTCTCATCGT
COL1A1	TGTGCGATGACGTGATCTGTGA	CTTGGTCGGTGGGTGACTCTG
COL3A1	CAAATAGAAAGCCTCATTAGTCC	GCATCCTTGGTTAGGGTCA
GAPDH	TTGGCCAGGGGTGCTAAG	AGCCAAAAGGGTCATCATCTC
