# Supplementary material for: Single-cell RNA-seq reveals fibroblast heterogeneity and increased mesenchymal fibroblasts in human fibrotic skin diseases
Source: Nat Commun. 2021 Jun 17;12:3709. doi: 10.1038/s41467-021-24110-y (PMC8211847; doi:10.1038/s41467-021-24110-y)
Supplement: Supplementary file 3 — Description of Additional Supplementary Files [file 41467_2021_24110_MOESM3_ESM.pdf]

Description of Additional Supplementary Files

Supplementary Data 1. Different expression genes between one fibroblast subpopulation and other fibroblast subpopulations.

Supplementary Data 2. Different expression genes between mesenchymal fibroblast subpopulation and other fibroblast subpopulations in keloid.

Supplementary Data 3. Others.vs.CD266+CD9- _differerent_expression_gene.

Supplementary Data 4. Different expression genes between sC1 fibroblast and sC4 fibroblast.
