# Supplementary material for: Single-cell RNA-seq reveals fibroblast heterogeneity and increased mesenchymal fibroblasts in human fibrotic skin diseases
Source: Nat Commun. 2021 Jun 17;12:3709. doi: 10.1038/s41467-021-24110-y (PMC8211847; doi:10.1038/s41467-021-24110-y)
Supplement: Supplementary file 8 — Reporting Summary [file 41467_2021_24110_MOESM8_ESM.pdf]

nature research

Corresponding author(s): Bin Yang

Last updated by author(s): May 23, 2021

Reporting Summary

Nature Research wishes to improve the reproducibility of the work that we publish. This form provides structure for consistency and transparency in reporting. For further information on Nature Research policies, see our Editorial Policies and the Editorial Policy Checklist.

Statistics

For all statistical analyses, confirm that the following items are present in the figure legend, table legend, main text, or Methods section.

n/a	Confirmed	
☐	☒	The exact sample size (*n*) for each experimental group/condition, given as a discrete number and unit of measurement
☐	☒	A statement on whether measurements were taken from distinct samples or whether the same sample was measured repeatedly
☐	☒	The statistical test(s) used AND whether they are one- or two-sided<br>*Only common tests should be described solely by name; describe more complex techniques in the Methods section.*
☒	☐	A description of all covariates tested
☐	☒	A description of any assumptions or corrections, such as tests of normality and adjustment for multiple comparisons
☐	☒	A full description of the statistical parameters including central tendency (e.g. means) or other basic estimates (e.g. regression coefficient) AND variation (e.g. standard deviation) or associated estimates of uncertainty (e.g. confidence intervals)
☐	☒	For null hypothesis testing, the test statistic (e.g. *F*, *t*, *r*) with confidence intervals, effect sizes, degrees of freedom and *P* value noted<br>*Give P values as exact values whenever suitable.*
☒	☐	For Bayesian analysis, information on the choice of priors and Markov chain Monte Carlo settings
☒	☐	For hierarchical and complex designs, identification of the appropriate level for tests and full reporting of outcomes
☒	☐	Estimates of effect sizes (e.g. Cohen's *d*, Pearson's *r*), indicating how they were calculated

Our web collection on statistics for biologists contains articles on many of the points above.

Software and code

Policy information about availability of computer code

Data collection	Single cell RNA sequencing data was collected by Novogene (Beijing, China) using Hiseq X for Illumina PE150 sequencing. Flow cytometry data was collected by BD FACSDiva™ software (v8.0.2). qRT-PCR data was collected by CFX Meastro software (V1.0).
Data analysis	Single cell RNA sequencing data was analyzed by 10× Cell Ranger package, Seurat R package (version 3.0.1), ARACNe-AP software, CellPhoneDB 2.0, destiny R package, and Monocle2. The code is available at Github: https://github.com/HobartJoe/Human_Keloid_scRNAseq. The code is also provided in Zenodo repository with the identifier (https://doi.org/10.5281/zenodo.4784648). Flow cytometry data was analysed by FlowJo (V10) software. qRT-PCR data was analysed by CFX Meastro software (V1.0).

For manuscripts utilizing custom algorithms or software that are central to the research but not yet described in published literature, software must be made available to editors and reviewers. We strongly encourage code deposition in a community repository (e.g. GitHub). See the Nature Research guidelines for submitting code & software for further information.

Data

Policy information about availability of data

All manuscripts must include a data availability statement. This statement should provide the following information, where applicable:

- Accession codes, unique identifiers, or web links for publicly available datasets
- A list of figures that have associated raw data
- A description of any restrictions on data availability

RNA-seq and scRNA-seq data have been deposited in the Gene Expression Omnibus (GEO) database under accession codes GSE175866 and GSE163973, respectively. All other relevant data supporting the key findings of this study are available within the article and its Supplementary Information files or from the corresponding author upon reasonable request. Source data are provided with this paper.

Field-specific reporting

Please select the one below that is the best fit for your research. If you are not sure, read the appropriate sections before making your selection.

- [x] Life sciences
- [ ] Behavioural & social sciences
- [ ] Ecological, evolutionary & environmental sciences

For a reference copy of the document with all sections, see nature.com/documents/nr-reporting-summary-flat.pdf

Life sciences study design

All studies must disclose on these points even when the disclosure is negative.

Sample size	Sample sizes were not predetermined based on statistical methods, but were chosen according to the standards of the field (at least three independent biological replicates for each condition). Required experimental sample sizes were estimated based on previous established protocols in the field. The sample sizes were adequate as the differences between experimental groups were reproducible. All n values are clearly indicated within the figure legends.
Data exclusions	In Fig. 2, the number of cells in sC8 and sC10-13 subpopulations was very small, so we did not include them in further analysis.
Replication	All experiments were repeated at least three times and all attempts at replication generated similar results.
Randomization	Human subjects used in this study were randomized. Randomization was not necessary for the other experiments because randomization was not required for these experiments based on previous experience.
Blinding	Blinding was not required in this study and investigators were not blinded to group allocation as all data in this study were analyzed equivalently within defined experimental groups.

Reporting for specific materials, systems and methods

We require information from authors about some types of materials, experimental systems and methods used in many studies. Here, indicate whether each material, system or method listed is relevant to your study. If you are not sure if a list item applies to your research, read the appropriate section before selecting a response.

Materials & experimental systems

n/a	Involved in the study	
[ ]	[x]	Antibodies
[x]	[ ]	Eukaryotic cell lines
[x]	[ ]	Palaeontology and archaeology
[x]	[ ]	Animals and other organisms
[ ]	[x]	Human research participants
[x]	[ ]	Clinical data
[x]	[ ]	Dual use research of concern

Methods

n/a	Involved in the study	
[x]	[ ]	ChIP-seq
[ ]	[x]	Flow cytometry
[x]	[ ]	MRI-based neuroimaging

Antibodies

Antibodies used	We describe all antibodies used in this study in the MATERIALS AND METHODS part of the manuscript.<br>1.mouse anti-ADAM12, Santa Cruz, Cat# sc293225, clone 1G3.<br>2.rabbit anti-NREP, Bioss, Cat# bs-0427R.<br>3.PE anti-human CD90, Biolegend, Cat# 328109, clone 5E10.<br>4.FITC anti-human CD9 Antibody, Biolegend, Cat# 312103, clone HI9a.<br>5.APC anti-human CD266, Biolegend, Cat# 314107, clone ITEM-4.<br>6.rabbit anti-collagen I, Abcam, Cat# ab34710.<br>7.rabbit anti-collagen III, Abcam, Cat# ab7778.<br>8.mouse anti-GAPDH, Proteintech, Cat# 60004-1-Ig, clone 1E6D9.<br>9.mouse anti-Periostin, AdipoGen, Cat# AG-20B-6000PF, clone OC-20.<br>10.mouse IgM Isotype Control, AdipoGen, Cat# ANC-290-810, clone TEPC 183.<br>11.rabbit anti-alpha smooth muscle actin, Abcam, Cat# ab124964.<br>12.Goat anti-Mouse IgG (H+L) Highly Cross-Adsorbed Secondary Antibody, Alexa Fluor 488, Thermo Fisher, Cat# A-11029<br>13.Goat anti-Rabbit IgG (H+L) Highly Cross-Adsorbed Secondary Antibody, Alexa Fluor Plus 555, Thermo Fisher, Cat# A32732
Validation	1.https://www.scbt.com/p/adam12-antibody-1g3?requestFrom=search<br>2.http://www.bioss.com.cn/prolook_03.asp?id=AF08169606000669&pro37=1<br>3.https://www.biolegend.com/en-us/products/pe-anti-human-cd90-thy1-antibody-4114

4.https://www.biolegend.com/en-us/products/fitc-anti-human-cd9-antibody-2212
5.https://www.biolegend.com/en-us/products/apc-anti-human-mouse-cd266-fn14-tweak-r-antibody-17153
6.https://www.abcam.cn/collagen-i-antibody-ab34710.html
7.https://www.abcam.cn/collagen-iii-antibody-ab7778.html
8.http://www.ptgcn.com/products/GAPDH-Antibody-60004-1-Ig.htm
9.https://adipogen.com/ag-20b-6000pf-anti-periostin-mab-blocking-oc-20-preservative-free.html
10.https://adipogen.com/anc-290-810-mouse-igm-isotype-control-tepc-183-preservative-free.html
11.https://www.abcam.cn/alpha-smooth-muscle-actin-antibody-epr5368-ab124964.html
12.https://www.thermofisher.cn/cn/zh/antibody/product/Goat-anti-Mouse-IgG-H-L-Highly-Cross-Adsorbed-Secondary-Antibody-Polyclonal/A-11029
13.https://www.thermofisher.cn/cn/zh/antibody/product/Goat-anti-Rabbit-IgG-H-L-Highly-Cross-Adsorbed-Secondary-Antibody-Polyclonal/A32732

Human research participants

Policy information about studies involving human research participants

Population characteristics	All the patients in this research were Han nationality. Keloid tissues were harvested during plastic surgery from three patients confirmed to have clinical evidence of keloid (Supplementary Table 1). All the keloids we used in this study were mature. No patient received chemotherapy, radiotherapy or intralesional steroids treatment prior to surgery. Normal scar tissues were obtained from three patients who underwent elective scar resection surgery (Supplementary Table 1). Keloids and normal scars were diagnosed on the basis of their clinical appearance, history, anatomical location and pathology.
Recruitment	Participants were recruited in Dermatology Hospital, Southern Medical University. We considered the characteristics of participants from experimental and control groups and no self-selection bias was present.
Ethics oversight	This study was approved by the Medical and Ethics Committees of Dermatology Hospital, Southern Medical University, and each patient signed an informed consent before enrolling in this study.

Note that full information on the approval of the study protocol must also be provided in the manuscript.

Flow Cytometry

Plots

Confirm that:

- [x] The axis labels state the marker and fluorochrome used (e.g. CD4-FITC).
- [x] The axis scales are clearly visible. Include numbers along axes only for bottom left plot of group (a 'group' is an analysis of identical markers).
- [x] All plots are contour plots with outliers or pseudocolor plots.
- [x] A numerical value for number of cells or percentage (with statistics) is provided.

Methodology

Sample preparation	Excised skin was immersed in physiological saline and then immediately transferred to the lab. The skin tissue was washed twice in PBS. After removal of the adipose tissue under the reticular dermis, samples were cut into 5 mm diameter pieces and incubated with dispase II (Sigma) for 2 h at 37°C. The epidermis was peeled off and discarded and the dermis was minced into small pieces and digested at 37°C for 2 hours using Collagenase IV (YEASEN, China). The resulting cell suspension was filtered through a 70μm cell strainer (BD Falcon), and centrifuged at 1500 rpm for 10 min. The supernatant was removed and the pellet was washed once with PBS at 1500 rpm for 5 min. The pellet was then resuspended in PBS+1% FBS for flow cytometry.
Instrument	BD FACSAria™ III Fusion cell sorters
Software	Collect: BD FACSDiva™ software. Analysis: FlowJo (V10) software.
Cell population abundance	Post-sort fractions were subjected to the same gating strategy during sorting and the abundance of the relevant cell populations was over 97%.
Gating strategy	First, select intact cells and remove small debris in FSC-A virsus SSC-A gating. The single cells were gated base on FSC/SSC and FSC-A/FSC-H. The viable cells were gated within DAPI negative gate, and fibroblasts were gated within CD90 positive gate. Mesenchymal fibroblasts were gated with CD9 negative/CD266 positive. The gating strategy was shown in Supplementary Fig. 6.

- [x] Tick this box to confirm that a figure exemplifying the gating strategy is provided in the Supplementary Information.
